# Development and palliative care staff reactions to a sleep regulation educational intervention

**DOI:** 10.1186/s12904-022-00902-x

**Published:** 2022-01-21

**Authors:** Elizabeth Capezuti, Rana Sagha Zadeh, Michael Ames Brigham, Brooke Ana Dias, Benjamin Chanhee Kim, Evie Lengetti, Belle Erikson, Nancy Swezey, Ana C. Krieger

**Affiliations:** 1 W.R. Hearst Foundation Chair in Gerontology, Hunter College, City University of New York (CUNY), 425 E. 25th Street, New York, NY 10011 USA; 2 Health Design Innovations Lab, Design and Environmental Analysis, Cornell University, 170 Martha Van Rensselaer Hall, Ithaca, NY 14853-4401 USA; 3 Human Ecology, Cornell University, Martha Van Rensselaer Hall, Ithaca, NY 14853-4401 USA; 4 M. Louise Fitzpatrick College of Nursing, Villanova University, 800 Lancaster Avenue, Villanova, PA 19085 USA; 5 Mount Sinai West Operating Room, New York, NY USA; 6 Departments of Medicine, Neurology and Genetic Medicine, Weill Cornell Medical College, Cornell University, 425 E. 61st St. 5th Floor, New York, NY 10065 USA

**Keywords:** Sleep, Circadian Rhythm, Non-pharmacological Intervention, Health Professional Education, Palliative Care, Acceptability, Usability

## Abstract

**Background:**

In palliative care, sleep and circadian rhythm problems are common symptoms. Nonpharmacological interventions are available; however, health care providers are not aware of these or lack the knowledge to effectively implement in practice. This study reports the content and design development of the PRIME™ (Program for Improving & Managing Environments for Sleep) sleep online educational intervention as well as the evaluation of the intervention by practicing nurses with a focus on perceived acceptability and satisfaction.

**Methods:**

Development of the education employed a multi-step process that assesses the current state of the science in this area (literature reviews), the needs of regional target recipients (hospice/palliative care staff), expert recommendations and views of a national pool of hospice/palliative workers. A cross-sectional, descriptive study with key staff informants evaluated the acceptability and usability of the modules using both scale-response items to rate the content and design of the modules and overall satisfaction and five open-response questions to suggest changes to the educational intervention.

**Results:**

Among 31 palliative care professionals, most rated the content and design favorably. A total of 20 participants provided suggestions to improve the educational intervention. Their comments were categorized into six themes: Integration into Practice; Content, Exercises and Material Provided by Modules; User Interface and Design; and Adapt and Expand Modules for Public, Family and Caregivers.

**Conclusions:**

The data suggest that the PRIME™ educational intervention can be an effective tool to train direct-care palliative care professionals on interventions for use in their daily practice. We also demonstrated that the educational intervention is feasible to deliver online and that the online modules appealed to respondents, suggesting that future delivery of the educational intervention can use the same or similar modes of presentation.

## Background

Sleep and circadian rhythm problems are common unmanaged symptoms burdening patients with advanced or life-limiting illness (es), as well as their caregivers and families [1, 2]. Individuals’ risk of sleep and circadian disturbance and their vulnerability to environmental and behavioral conditions to develop such symptoms increases with age, physical or cognitive impairments, pain and comorbidities [3]. Sleep and circadian rhythm disruptions are associated with worsening in pain and inflammation, development of depression and delirium, and increased risk for falls [4]. It also affects formal and informal caregivers, often leading to worsening sleep quality and burnout [2, 5–7].

Although evidence has shown that non-pharmacological interventions can be effective in reducing sleep and circadian disturbances [8, 9], organizational support to bring these practices to the bedside is unfortunately sparse [10], and most staff have reported not receiving formal education to manage these symptoms [11]. From previous work of authors, focus groups of multidisciplinary palliative care providers from three demographically diverse counties in New York State reported that “provision of educational interventions” was among the top recommendations to address sleep and circadian disturbances in individuals with advanced illness [12]. Other interventions referenced the use of environmental and technological resources, the multidisciplinary care team and organizational leadership. Focus group respondents highlighted the need for “up-to-date information on sleep, biology and circadian rhythms” to “optimize clinical decisions in order to frequently evaluate the effectiveness of care” and “to decide in an educated manner between non-pharmacological and pharmacological interventions, avoiding unnecessary pharmacological interventions and taking patient preferences into consideration.” Thus, they could individualize these interventions to meet the patient’s needs more effectively. Specifically, educating family members and patients on the importance of sleep hygiene, how to detect and manage sleep and circadian disturbance and ways to modify the sleep environment were also described as key unmet needs in palliative care practice. It is well documented in the literature that education is a necessary intervention but often not sufficient by itself as it has to be combined with other interventions (e.g. reminders, automation) for sustainable and sizeable benefits [13].

In response, our team is developing a multisystem intervention entitled PRIME™ (Program for Improving & Managing Environments for Sleep), a complex sleep health intervention [14], that includes an educational component on management of the patient’s intrinsic state, as well as the situational context (e.g. care and environment), supplemented by a technological component [15]. The two components act both independently and interdependently. Prototypes for the PRIME™ technological component was developed and evaluated from 2013 to 2016, functioning as an environmental and behavioral monitoring and feedback system of an individual patient’s sleep. The PRIME™ educational component is meant to enhance nursing staff’s knowledge of causes of sleep disruption, assessment techniques and various non-pharmacological interventions that can be tailored to individual patients’ needs. This module is a key component that allows for proper use of the monitoring technology by providing education and training [16].

Many health provider educational interventions are based solely on scientific literature and expert opinion. They frequently do not work in practice because they fail to capture the current and specific learning needs of the target population, including real life experiences at the bedside as well as unique patient conditions [17]. Thus, we integrated the input of direct care providers during several stages of the educational intervention’s development. The development process followed the Medical Research Council (MRC) Framework for the Development and Evaluation of RCTs for Complex Interventions to Improve Health [18–20]. Accordingly, the designing of interventions entails iterative cycles of development with stakeholder input throughout the process, while acknowledging the relevance of the practice context [21].

This study reports the content and design development of the PRIME™ sleep educational intervention as well as the evaluation of the intervention by practicing nurses with a focus on perceived acceptability/ satisfaction and views of the likely effectiveness of the educational intervention on practice.

## Methods

### Content Development

We employed a multi-step process for the development of the PRIME™ educational intervention. This included assessing the current state of the science in this area (literature reviews), the needs of regional target recipients (hospice/palliative care staff), expert recommendations and views of a national pool of hospice/palliative workers.

#### Literature Reviews

Two systematically-conducted literature reviews of non-pharmacological sleep interventions for adults with advanced serious illness across settings [22] and adults receiving care in institutional settings [8] informed content areas of the educational modules. These reviews identified interventions that improved sleep for persons receiving palliative care in various settings; however, each demonstrated either moderate or weak efficacy.

#### Focus Groups

We then conducted a multi-facility study that gathered care providers’ bedside experiences and insight via focus groups. Content derived from our prior review of the literature was summarized in a focus group guide that we employed with 32 hospice/palliative care staff from four organizations in three counties of northern New York State [12]. We asked how they manage sleep and circadian disruptions of terminally ill persons and what they think might enhance sleep in this population. Respondents echoed many of the interventions identified in the systematic reviews while also indicating a need for general sleep education including pathophysiology, assessment and sleep needs of those with complicated illnesses such as cancer and dementia. Respondents also expressed a desire for content related to individualizing care and providing education for family and patients.

#### National Survey and Expert Professional Panel

The themes and concepts were then further validated via a national survey. The survey tool was adapted from another study designed to gauge healthcare providers’ familiarity, knowledge and application of sleep assessment instruments and interventions for persons with dementia [23]. Additional items were added based on the findings from the literature reviews and focus groups. The survey aimed to identify healthcare professionals’ use and perceptions of interventions to promote sleep among adult patients receiving palliative/hospice care as well as their interest in sleep education. We established content validity and item clarity with six palliative healthcare professionals with an average of 5.1 (SD=3.9) years of inpatient and/or outpatient experience in large, academic-affiliated health systems in New York City [24].

The survey asked respondents to rate (as tried/recommended and feasible in their practice setting) 36 individual sleep promoting interventions as well as indicate their educational needs regarding sleep. Following approval by the CUNY IRB, the Hospice and Palliative Care Nursing Association and the Gerontological Advanced Practice Nurses Associations, a URL link to the online, anonymous survey was sent to potential respondents by blast emails. We also posted an invitation and URL link on Facebook and LinkedIn groups related to palliative/hospice care. We received non-identifiable data from a non-random, purposive sample of 108 registered (43.5%) or advanced practice nurses (56.5%) working mostly in palliative care inpatient (40.5%) or outpatient (50%) settings and representing all US regions [24].

The topics chosen by more than 40% of respondents in order of magnitude were: sleep physiology in dementia, bundling sleep treatment with fatigue and depression, sleep physiology in cancer, complementary and alternative health therapies, standardized tools for evaluating sleep and sleep hygiene measures and environmental assessment of factors that influence sleep. The respondents indicated that nearly 75% (27 out of 36) of the interventions would be feasible in their work environment, and these were included in the educational modules with the associated evidence level. Respondents indicated a preference for web-based learning modules (i.e. brief videos) and PDFs of educational intervention summaries.

### Design and Development of the Educational Intervention

The survey findings, together with the literature reviews, focus group results, expert panel recommendations and evidence-based sources for topical content were synthesized into 8 scripts by a nurse educator (EC) and a graduate student in nursing (NS) and then reviewed for accuracy by a physician sleep specialist (AK). Two faculty in a college of nursing (EL, BE) developed objectives for each module and the overall course and created four to eight test questions per module based on those learning objectives; respondents answer these questions prior to viewing the module and once again after viewing [25].

An instructional designer was then consulted to convert the content into eight 18-minute, narrated online modules. The content covered in the 8 modules is listed in Table 1. Guided by the learning objectives, the designer reformatted the content experts’ script into compelling content for online learning. The designer created instructional graphics and embedded photos to enhance the visual reinforcement of the content. Visual components, including choice of images and infographics, and method of delivery of content via text and audio were then reviewed by a nurse educator (EC), design researcher (RZ), sleep specialist (AK) and graduate student in environmental psychology and human factors (MB). A learning technology consultant deployed these modules on an e-learning platform (Moodle) paired with a project landing page, allowing respondents to easily access the modules on the Internet and seamlessly connect to test questions and the final evaluation survey.

**Table 1:** PRIME™ Sleep Education Intervention Topics and Content Areas

Topic	Content Areas
Sleep Basics: General Physiology	Normal sleep Physiology of sleep onset Phases of sleep Significance of sleep to overall health Endocrine and neurologic physiology of sleep Sleep wake regulation
Sleep: Screening & Assessment	Sleep/sleep hygiene screening Psycho-social, cognitive and mental issues Assessment of specific sleep problems When to refer to sleep specialist
Sleep: Dementia	Sleep and brain health Sleep problems by dementia types Caregiver sleep problems Treatment considerations Safety precautions
Sleep: Cancer	Risk factors for sleep disturbances Cancer treatment associated sleep disturbances Symptom clusters Consequences of sleep disturbances in cancer patients Treatment considerations
Sleep Intervention: Overview	Pharmacological interventions Side effects of sleep medications Daytime social and physical activities Nighttime clinical care processes
Sleep Hygiene	Nighttime sleep habits Exercise and sleep Psychotropic substances and sleep Stimulus control and sleep restriction
Sleep Environmental	Lighting Auditory environment Ambient temperature
CHP MBP Interventions	Complementary and alternative practices Mind-body practices to improve sleep

### Evaluation

To evaluate the feasibility of adopting these modules in a professional setting, we conducted a cross-sectional, descriptive study with key staff informants to evaluate the acceptability and usability of the modules. The educational specialists developed a survey that asks respondents for an assessment of module content and presentation at the completion of course; this survey was developed in Qualtrics software (Version 1.3; Provo, Utah) and linked to the module e-learning platform. The survey link was automatically made accessible after a participant completed all modules. An evaluation of the module content’s effectiveness in increasing knowledge will be discussed in a forthcoming publication.

#### Site/Respondents

The study was implemented at a non-profit organization that offers inpatient and community-based palliative care services to patients with any advanced illness and operates offices in two counties in Central New York State. Both offices are the primary provider of palliative care for the populations residing in those two counties. The modules were developed for licensed nurses originally; however, the agency requested that all staff be provided the opportunity to participate. Sixty employees—including clinical, supportive services and administrative staff—were invited to participate in the study by email from the agency administrators. Although viewing the modules was highly encouraged in the email, the employees were informed that participation in our study, the evaluation component, was voluntary. Thirty-one employees (51%) completed the study; four employees completed more than one module but less than eight, and their results are not included in this analysis.

#### Measures

Investigators developed an evaluation tool consisting of 19 items (11 scale-response items, five open-response questions and three single-choice demographic questions). The 11 scale-response items asked respondents to rate the content and design of the modules and their overall satisfaction with the educational intervention on a 4-point Likert type scale (i.e. strongly agree, agree, disagree or strongly disagree). The five open-response questions asked respondents to suggest changes or additions to the modules and asked which strategies they may plan to incorporate into their practice. A list of all items can be found in Table 1.

#### Procedures/Human Subjects

Cornell University Institutional Review Board and the CUNY Human Research Protection Program approved the study protocol. In the spring of 2019, members of the research team visited each county office and provided a brief, in-person introduction to the project during a staff meeting. The research team described the consent process and answered any questions regarding the research or their rights as study respondents. The organization provided the research team with a list of staff email addresses used to generate Moodle user accounts, allowing the respondents to access the online consent form, educational modules and evaluation survey. Upon logging in, respondents that consented had access to all eight modules and could watch them in any order. Once all eight modules and accompanying knowledge assessment questions were completed, a link to the final evaluation survey was unlocked.

Respondents were instructed to complete the modules at their convenience and were informed prior to participation that an incentive will be provided. Following the completion of the final evaluation survey, the respondents received an email link for a monetary gift card of $100; registered nurses who participated in the study received 7.4 contact hours through Villanova University Continuing Education Program in Nursing and Healthcare.

#### Data Analysis

Deidentified evaluation data were downloaded into SPSS (Version 26; Armonk, NY: IBM Corp.) in which basic descriptive statistical analyses were performed. Responses to open-ended questions were analyzed using qualitative content analysis [26]. All data were initially coded first (BK) for the development of the preliminary coding schema (themes and subthemes) emerging inductively from the responses. A preliminary coding schema was then revised by the entire team, and a sample of quotes were paired with each theme and subtheme (EC, RZ, BD, MC). After the coding schema was finalized, the full dataset was then coded again by two independent reviewers (BK & MB) which had inter-rater reliability of over 90%. Any conflicts were reviewed by a third reviewer (RS) and discussed with the entire team for consensus on a final coding schema.

## Results

A total of 31 participants completed all eight educational modules and the evaluation. Most were licensed nurses (54.8%, *n*=17) or nursing assistants (25.8%, *n*=8) while others included social workers (12.9%, *n*=4) and administrators/managers (6.5%, *n*=2). All participants worked at one of the two offices of the participating palliative care agency providing home-based (51.6%, *n*=16), in-patient residential (32.3%, *n*=10), nursing home-based (22.6%, *n*=7) and/or out-patient (19.4%, *n*=6) palliative care services. Most (54.8%, *n*=17) worked in palliative care for greater than 6 years while 22.6% (*n*=7) had 4 to 6 years, 16.1% (*n*=5) had 1 to 3 years and 6.5% (*n*=2) had less than one year of experience.

### Quantitative Findings

Table 2 summarizes the care providers’ evaluation of the survey regarding the content and the design of the educational intervention favorably with 10 of 11 questions receiving at least 90.3% responses of “agree or strongly agree.” The highest evaluation was for the question “provided content that I can use to address my patient's sleep issues” which scored 100% satisfaction for all participants (*n*=31); the lowest evaluation referenced the visual design, which was positively scored by 80.6% of participants.

**Table 2:** Palliative Care Staff Evaluation (*n*=31)

#	Survey Question	% Strongly Agree/Agree
1	have clear learning objectives	100% (31)
5	provided content that I can use to address my patient's sleep issues	100% (31)
4	provided information about sleep that I did not know before	96.8% (30)
3	had questions which were relevant to the modules’ content	93.5% (29)
6	will enhance my ability to communicate information about sleep to my patients and/or their families?	93.5% (29)
8	the quality of the voice (rate of speed, word pronunciation) of the voice was good and easy to understand	93.5% (29)
10	I would recommend these educational modules to a colleague	93.5% (29)
9	the length of the module was just right	90.3% (28)
11	I am very satisfied with the learning experience	90.3% (28)
2	provided the right amount of detail	90.3% (31)
7	the visual design (background, illustration, photos, tables and figures) enhanced the presentation	80.6% (25)
	Overall	93.0%

### Qualitative Findings

A total of 20 participants (64.52% *n*= 31) provided responses to the open-ended questions. Table 3 displays the responses synthesized into six themes and 15 subthemes. The themes in descending order of prevalence are Integration into Practice; Content, Exercises and Material Provided by Modules; User Interface and Design; and Adapt and Expand Modules for Public, Family and Caregivers.

**Table 3:** Categories and Subcategories of Participant Open-Ended Responses

Themes	Subthemes		No. of Related Responses	Total No. of Responses
Integration into Practice	Affirmation that the Modules Will be Useful in Practice		21	40
	Actions Taken to Integrate into Current Work Processes	Sleep Hygiene	8	
		Aromatherapy	3	
		Disease-Specific	3	
		Environment	3	
	Other		2	
Content, Exercises and Material Provided by Modules	Improvements to the Content, Exercises and Material Provided by Modules	Modules	15	35
		Quiz Questions	2	
	Content, Exercises and Material Provided by Modules were Sufficient/Good		14	
	Level: Too High or Too Low		4	
User Interface and Design	Pace, Rate of Delivery		6	25
	Engagement and Interactivity		5	
	Length/Time Taken to Complete the Modules		4	
	Overall Aesthetics and Visuals		4	
	Design was Sufficient/Good		3	
	Software Improvements		3	
Adapt and Expand Modules for Public, Family and Caregivers	Develop Education for Public/Family		3	7
	Need Resources i.e. Handouts, Transcripts		4	

#### Integration into Practice

A total of 17 out of 20 respondents expressed that the knowledge gained from the educational modules was very useful in practice (21 comments). Our analysis of participant feedback revealed that the education program offered benefits in the following eight topics with exemplar quotes:

Increasing participants’ awareness of causes of sleep disruption, assessment techniques and non-pharmacological interventions and to improve their care practice.

Empowering participants to meet their patient disease-specific needs;

Equipping them with information on a variety of possible factors (e.g. light, noise, aromatherapy, melatonin and medication adjustments) to address and alleviate patient suffering especially with awareness to no cost or low-cost solutions (e.g. natural light or impact of blue vs red light in the evening);

Providing awareness on simultaneous implementation of multilevel integrative solutions as opposed to an individual solution only (e.g. medication) and made a set of options available to customize the solutions to the patient individualized needs;

“Each pt is different and what works with one does not work with another. I will be utilizing suggestions to find the right fit for each pt.,” said a registered nurse*.*

Increasing their trust in alternative approaches (e.g. aromatherapy);

Enriching their practical knowledge and vocabulary to better educate the patient and family;

“It has helped me understand, and give [s] me terminology, to assist pts and family with self care in regards to their sleep quality,” said a nursing assistant*.*

Initiating self-efficacy;

“I am glad sleep is finally being one of the key components in anyone's mental and physical health [assessment] … This has certainly enhanced my knowledge for my own sleep hygiene and to help others. Thank you,” said this registered nurse*.*

Becoming more attentive to catering to disease-specific subpopulations due to the knowledge gained on multiple factors that may affect the sleep-wake cycle of oncological or hospice patients, and persons with cognitive impairment.

Several articulated in their survey response (17 comments) they are taking actions to integrate the educational material (e.g. aromatherapy, environment and sleep hygiene solutions) into their day-to-day work processes or use the obtained knowledge to evaluate their patient’s condition.

“Yes, I will be making suggestion regarding the use of lavender and also suggestion limiting blue light (internet use) in bed when unable to sleep,” said a registered nurse*.*

#### Content

There were a total of 35 comments from 20 participants about the content. Participants provided feedback in the following areas:

The content was informative, satisfactory, sufficient and well suited for their level of education (14 comments);

The level of the content was too “high” or too “low” (i.e. too difficult to be comprehended) (4 comments);

And needed improvements (17 of comments): the incorporation of new, specific, and/or narrowed educational material and tools such as more reflective questions to help retain the message and supporting content for those at different sleep knowledge education levels.

“Some of the information in the modules was over my head-there were details that were too scientific and a bit difficult for me to understand. Perhaps those with more education under their belt had an easier time comprehending some of the information. However, most of it was not too difficult and easy to follow. I learned a lot,” said a registered nurse*.*

The disease-specific modules (e.g. dementia, cancer, etc.) were found helpful for meeting patient individualized needs; participants felt a need for more disease-specific modules as well as population-specific ones (e.g. age group). Additionally, one registered nurse asked for additional training on indications and contraindications of sleep medication, especially for end-of-life patients.

Other registered nurses provided specific content expansion such as going into more detail about how “medications and side-effects impact sleeping patterns” and including information about circadian rhythms, needing more information pertaining to the “the effects of nicotine on sleep,” and the relationship between snoring and “the effect on sleep as well as interventions to improve quality of sleep in a [patient] who is snoring.”

#### User Interface and Design

When asked about the visual components and delivery of the education, participants suggested making the educational content more interactive with incentives to continue to learn and retain information (5 comments).

“Having intermittent questions/something interactive mid-way in the module would encourage me to watch every minute of the module and not skip through,” stated a social worker*.*

In terms of visuals (4 comments), participants appreciated the combined audiovisual delivery. A few shared there were opportunities for material to be more colorful and engaging to help maintain attention throughout the training. Participants found the use and diversity of the photos agreeable and asked for increasing diversity of graphic data presentation.

Participants’ feedback on the pace of audio varied. The pace was perceived as too fast, too slow, or just right with positive recognition of the voice-over (6 comments).

Further, participants commented on the length of the modules (4 comments).

A registered nurse shared that the modules were “Short and colorful [and] lively”*.*

*“Modules are too long, too much information for one section,” said a registered nurse*.

Participants also stated that they believed the design was sufficient and/or good (3 comments) and some provided software improvement recommendations that will be useful for future revisions (3 comments).

#### Adapt and Expand Modules for Public, Family and Caregivers

A range of great ideas in 7 comments was shared pertaining to the expansion of the educational intervention to tailor more varied stakeholders or retain the materials for the same users. Several participants emphasized a need for educational modules for other stakeholders (e.g. family caregivers and the general public). Others offered ideas about how the content shared could be adapted to other populations (3 comments).

*An LPN detailed that “If th [is program] was shortened [it] would be a good tool for family caregivers*.”

“I hope these are available for families to even watch so they can understand and help their loved ones,” said a nursing assistant*.*

Others recommended supplemental resources such as handouts, in-person training, and transcripts to help retain and reinforce knowledge (4 comments).

## Discussion

The Medical Research Council framework informed the process that we used to develop the educational intervention and evaluate its acceptability and usability. This iterative development process, with input from stakeholders, resulted in preliminary support for online patient sleep promoting education targeting for palliative care professionals. The unanimous affirmation from participants that the modules provide content to help them address patient sleep issues suggests that the PRIME™ educational intervention can be an effective tool to train direct-care palliative care professionals on interventions for use in their daily practice.

The analysis of commentary indicated that the educational intervention provided participants with the knowledge to meet their patients’ disease-specific and individualized needs, address and alleviate patient suffering (especially through no and low-cost solutions), implement multilevel solutions, utilize alternative approaches (e.g. aromatherapy), tailor to specific subpopulations and improve their own wellness. For a majority of participants, the program was found to provide enriching, practical knowledge and vocabulary to better educate the patient and family, and several stated (17 comments) they have actionable plans to integrate the knowledge into practice.

The range of satisfaction level with the content demonstrated the importance of equalizing knowledge among healthcare professionals. One participant was not satisfied and found the information to be well known; whereas others stated it was challenging but provided new information and a few thought the level of the content was just right (14 comments). Participants expressed the need for content improvements (e.g. more disease-specific modules and adaptations for all education levels) and supportive materials (e.g. transcripts and training) for further learning opportunities. The participants’ commentary allowed us to analyze the variation and conclude that sleep knowledge competency was heterogeneous across participants and future development of the educational intervention should take this into account.

The user interface design was discussed in terms of visuals, pace, voice length and instructiveness. Participants indicated the need for increasing engagement and additional appealing visuals—such as the use of more color and different forms of graphic displays of quantitative data—to keep their attention. However, there was expressed approval for photo diversity. The perception of the module speed and length were diverse: comments on both ends of the spectrum and statements of approval.

We utilized an experienced instructional designer who incorporated a unifying color and circadian-themed (reflecting the 24-cycle in 3-hour intervals for each of the 8 modules) format as well as photos that reflected diverse nursing and patient/caregiver populations. 19.4% of participants, however, indicated that the visual design of the modules did not enhance their learning.

We did not, as several participants noted, provide interactive features such as self-tests throughout the module (with explanations of the right and wrong answer). Learner feedback is known to reinforce learning as well as more actively engages the learner [27]. The open-ended responses provided valuable feedback into the perspective of key stakeholders on how to meaningfully revise and expand the modules for subsequent iterations. Specific content recommendations reflect the participants’ interest in more in-depth information, especially for enhancing the online educational content with downloadable handouts. Others have noted that learners prefer such resources to reinforce and sustain learning [24]. In response, the team is developing a one-page summation to accompany each module.

We also demonstrated that the educational intervention is feasible to deliver online and that the on-line modules appealed to respondents, suggesting that future delivery of the educational intervention can use the same or similar modes of presentation. This is consistent with the national trend for continuing nursing education delivered on-line since the learner reviews at their own pace and schedule and can focus on content based on their individual needs [24]. There is little or no difference in e-learning compared to traditional lectures in regard to changes in staff knowledge, behaviors or patient outcomes [28].

Another area for development is the creation of educational modules and written guides aimed toward the general public and those with advanced serious illness and their family/friend caregivers. Most research concerning patient/caregiver sleep education utilizes in-person individual [29] or group instruction [30] and/or written materials [27]; thus, we will likely add a module for staff on educating patients and caregivers that can facilitate communication and reinforce patients’ and caregivers’ online learning.

In a separate paper, we analyze the effect of the modules on knowledge acquisition. Education alone, however, does necessarily improve practice [31]. It requires active learning [32] as well as ongoing clinical and organizational support to assist with embedding implementation into practice [33]. As indicated in a national survey, palliative care nurses are interested in the use of non-pharmacological interventions but it depends on their organizations’ openness and their provision of these alternative sleep-promoting strategies [24]. Thus, we plan to pilot the full PRIME™ intervention (monitor and education including additional interactive features) to examine its relative efficacy on changing staff practices and improving sleep outcomes for those with advanced illness.

### Limitations

We used a one-time online evaluation questionnaire but would likely receive more specific feedback if we tested the educational design by conducting a user interface or “think aloud” evaluation [34]. Additionally, the respondents are only a sample from one region in upstate New York and, thus, are not representative of all palliative care staff. We intended the modules for licensed nurses; however, they represented just over half of the participant cohort.

Nurses are not the only stakeholder group which implies that future versions should be designed to accommodate those without a clinical background (e.g. patients and family members/caregivers). The next stage of intervention testing will take into account the views of intervention effectiveness from these stakeholders as well as direct care staff. High acceptance scores could also be attributed to participants being (1) volunteers and (2) incentivized with a gift card. In future research, we will seek a larger sample size to further confirm our findings for the intervention to be generalized to a broader population of palliative care professionals.

## Conclusions

Sleep quality has important implications for the overall quality of life among those with advanced serious illness. Although disturbed sleep is a common symptom among those receiving palliative care services, it may be either poorly managed or only treated with medications with a high side-effect profile. Results from this study support further testing of the PRIME™ educational intervention in improving patient sleep management by palliative care professionals. Additional investigations are essential to allow for the generalizability of these results. These forthcoming studies should include data collection from palliative care staff in various palliative care settings and geographic locations with content expansions to address specific learning needs of non-nurse staff, patients and family caregivers. Importantly, further research is needed to validate behavior change in nurses and other direct care staff and palliative care patients’ sleep outcomes.

## Data Availability

The datasets used and analyzed during the current study are available from the corresponding author on reasonable request.

## Abbreviations

PRIME: Program for Improving & Managing Environments for Sleep
MRC: Medical Research Council
LPN: Licensed Practical Nurse
CUNY: City University of New York
CHP MBP: Complementary and alternative practices, Mind-body practices
